# The Influence of Easing COVID-19 Restrictions on the Physical Activity Intentions and Perceived Barriers to Physical Activity in UK Older Adults

**DOI:** 10.3390/ijerph191912521

**Published:** 2022-09-30

**Authors:** Jason Tallis, Darren L. Richardson, Emma L. J. Eyre

**Affiliations:** Research Centre for Sport, Exercise and Life Sciences, Coventry University, Coventry CV1 5FB, UK

**Keywords:** ageing, COM-B, COVID-19 recovery, healthy ageing

## Abstract

COVID-19 has had profound effects on physical activity behaviours of older adults, and understanding this impact is essential to driving public health policies to promote healthy ageing. The present study aimed to determine; (1) intended physical activity behaviours of older adults following the easing of UK COVID-19 restrictions; (2) the relationship between self-reported physical activity and intended physical activity behaviour; (3) perceived barriers to achieving the intended physical activity goal. Ninety-six participants (74.8 ± 4.4 years; 52 female) from a longitudinal study examining the impact of COVID-19 on physical activity were recruited. Participants outlined their future physical activity intentions and completed the COM-B Self Evaluation Questionnaire. Participants were split into groups based on their intention to ‘Maintain’ (*n* = 29), ‘Increase’ (*n* = 38) or ‘Return’ (*n* = 29) to pre-COVID-19 physical activity. Self-reported physical activity undulated over the pandemic but was mostly equivalent between groups. Intended physical activity behaviour was independent of self-report physical activity. Capability and motivation factors were the most frequently cited barriers to the intended physical activity behaviour, with a greater number of capability barriers in the ‘Return’ group. Such barriers should be considered in the COVID-19 recovery public health physical activity strategy for promoting healthy ageing.

## 1. Introduction

Globally, populations are ageing [1], and whilst people are living longer, increasing age is associated with a growing number of comorbidities [2] and gains in health expectancy fail to match gains in life expectancy [3]. Whilst increasing age not only influences health, wellbeing and quality of life, it also results in substantial economic and societal costs [4]. Physical activity (PA) has a pivotal role in promoting healthy ageing [5], with well-established links to improved cardiovascular and muscle function, psychological health and wellbeing, and reduced prevalence or severity of disease [6]. Despite the well-known benefits of PA, adults over the age of 75 years represent the least physically active population in the United Kingdom (UK) [7], with sedentary behaviour being further compounded by government restrictions and anxieties as a result of the global COVID-19 pandemic [8]. As we move towards a strategy of living with COVID-19, it is important to evaluate how the COVID-19 pandemic has affected the PA intentions of older adults, but also the perceived barriers and enablers to the target behaviours in order for stakeholders to better strategize interventions to promote and facilitate PA in this population.

**Figure 1:**
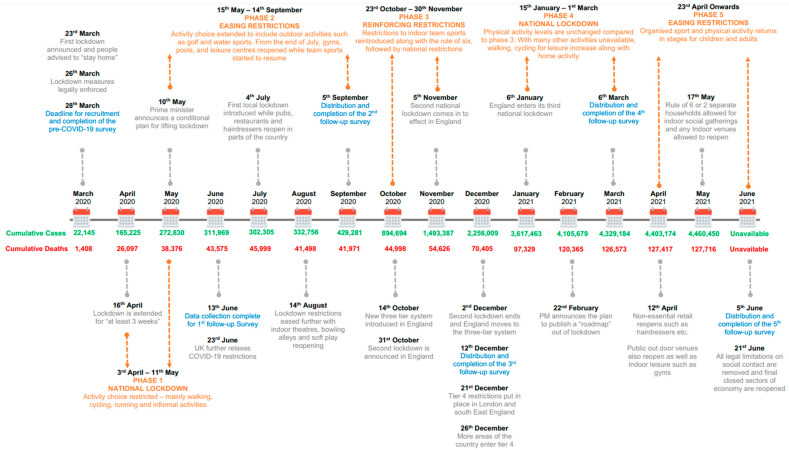
Overview of COVID-19 events in the UK between March 2020 and June 2121. Reprinted from [14].

A plethora of published work has evaluated the effects of the ongoing COVID-19 pandemic on the PA behaviour of older adults, with a consensus that a combination of both government imposed restrictions on movement and social interaction, in combination with anxiety related to COVID-19 illness, generally resulted in a reduction in the frequency of PA and an increase in sedentary behaviours in older adult populations (See review [8]). In particular, the initial period of lockdown in England resulted in a 27% increase in older adults that were inactive (completed <30 min activity per day), a 39% reduction in in the duration of strength and balancing activities, with modelling predicting an up to 6% increase in total falls and an additional £211 million cost to the health and social care system over the next two and a half years [9]. Given that older adults are amongst the least physically active populations [7], such changes in PA and sedentary behaviour likely exacerbate the risk of an unhealthy ageing trajectory. Even short-term reductions in PA in older adults have been shown to reduce lean mass, evoke anabolic resistance and reduce muscular strength [10,11], which may influence perceived ability, confidence and competency to increase or even return to pre-pandemic PA behaviours [12,13]. However, given the extended period of undulating government imposed restrictions in the UK (Figure 1), the potential for long term changes in PA behaviour is likely compounding the impact of restrictions on confidence and competency to increase or return to pre-COVID-19 PA behaviours.

Intuitively, promoting a physically active lifestyle has been shown to be an effective intervention to promote healthy ageing [5] and is an essential aspect of a healthy ageing policy. Physically active older adults are at reduced risk of all-cause mortality, cardiovascular mortality, cancer, falls, cognitive decline and activities of daily living (ADL) disability [1]. Despite the well-known positive outcomes of increased PA, the public health challenge of sedentary behaviours is rooted in behaviour change psychology, where there is a need to promote engagement with PA and sustainability of exercise behaviour. Understanding the barriers and enablers to PA that are specific to older adults are essential to provide targeted interventions to support behaviour change and such understanding is essential in the design of a healthy ageing policy. Whilst there is a growing body of evidence that has evaluated the perceptions of older adults [15,16,17,18], some specific to the UK older adult population [19,20], evolution in social-economic challenges means that evaluation of barriers and motivators for PA should be a continued area of focus. In recent times there has been no bigger population level impact on PA than that imposed by COVID-19. As the global population learns to live with COVID-19 and people of the UK specifically, come to a period of easing all legal restrictions, understanding the PA intentions of older adult populations and the perceived barriers and enablers to PA provides essential information to stakeholders planning post-COVID-19 health strategies.

Behaviour change theory should be embedded into PA interventions to increase the likeliness of success [21]. Whilst there are several behaviour change theories, each with their own strengths and limitations [22,23], the Behaviour Change Wheel (BCW) provides a synthesis of many previously established frameworks [24]. Central to the BCW is the Capabilities, Opportunities, Motivations, Behaviour (COM-B) model [24] which is recognised by the National Institute for Health & Care Excellence as a key framework for understanding and supporting behaviour change [25]. With this in mind, the present study aimed to (1) determine the intended PA behaviours of older adults following the easing of COVID-19 restrictions in the UK; (2) understand the relationship between self-reported PA and intended PA behaviour; (3) utilise the COM-B Self Evaluation Questionnaire to identify capability, motivation and opportunity barriers to engage in the intended PA behaviour.

## 2. Materials and Methods

Following institutional ethics approval (P105110) and informed consent, 96 participants (52 female), aged 74.8 ± 4.4 years were recruited from a longitudinal online survey-based observational study (that took place between March 2020 and July 2021) examining the impact of COVID-19 on PA, perceived physical function and mood [14]. An initial pre-COVID-19 lockdown survey was completed (retrospectively where necessary as the first lockdown commenced on 23 March 2020). As part of our previous work [14], self-report PA data, was collected at 3-monthly intervals (Figure 1) using the International Physical Activity Elderly [26] and has been analysed to fulfil the experimental aims of the present study. From the International Physical Activity-Elderly questionnaire (IPAQ-E), Metabolic Equivalent of Task (MET-minutes) of PA during the prior seven days were calculated at each time point using recommended methods (www.ipaq.ki.se, accessed on 1 February 2020). The IPAQ-E was selected as it has good validity and reliability for measuring the PA of older adults [27].

At the final sampling point (July 2021), which coincided with the easing of COVID-19 restrictions in the UK, participants were asked to outline their future PA intentions and complete the COM-B Self Evaluation Questionnaire [24] to provide information on the perceived barriers to their desired PA behaviour. Evidence supports the acceptability, validity, and reliability of the COM-B Self Evaluation Questionnaire for self-evaluating capabilities, opportunities, and motivations [28]. 

The COM-B Self Evaluation Questionnaire was prepared and distributed using JISC online surveys (https://www.onlinesurveys.ac.uk, accessed on 3 April 2021). At the outset, participants were asked to select one of four options that best represented their goal/intention for their PA level as COVID-19 restrictions eased. Participants were able to select from the following statements, ‘Maintain the physical activity practices I developed during COVID-19 going forward’, ‘Try to increase physical activity levels as restrictions ease’, ‘Return to my physical activity levels prior to COVID-19 restrictions’ or ‘Other [PA intention]’. As such, participants were split into three distinct groups, ‘Maintain’ (*n* = 29), ‘Return’ (*n* = 29), ‘Increase’ (*n* = 38). With respect to their PA intention, participants were then asked to select which of the 22 statements (if any) linked to three separate categories, ‘Capability, Opportunity, and Motivation’ best represented the challenges to achieving the desired behavioural outcome. If an item was selected, participants were provided with an opportunity to provide further written detail if necessary. 

### Data and Statistical Analysis

Statistical analysis was performed using SPSS 26.0 (Chicago, IL, USA). Statistical significance was a priori set at an alpha level of *p* < 0.05. Graphical presentation was performed using GraphPad Prism (Version 8.3.1, San Diego, CA, USA).

A Group X Time mixed model ANOVA was performed to evaluate potential differences in self-report PA between the groups. Due to violation of normality, data transformations were performed and normality was rechecked using the Kolmogorov–Smirnov test. Square root transformation was the most effective at improving the data distribution, and as such, was subsequently used in the analysis. On a small number of occasions, the data were not normally distributed, however, ANOVA is robust to violations of normality [29,30]. Violations of Sphericity were corrected using Greenhouse–Geisser and Bonferroni pairwise comparisons were used to explore significant main effects. Eta squared (*η*^2^) was calculated as an estimate of effect size and was interpreted as small (>0.01), medium (>0.06) or large (>0.14) [31]. For pairwise comparisons, Cohen’s d was calculated and corrected for bias using Hedge’s *g* [32]. Hedges *g* effect size was interpreted as trivial (<0.2), small (<0.6), moderate (<1.2) or large (>1.2) [33]. To confirm that this approach was robust given the violations of normality, a series of Kruskal–Wallis tests were performed on the pre-transformed data in order to evaluate between group differences in PA at each of the measured time points.

Ordinal logistic regression was performed to establish if self-reported PA determined intended PA behaviour. PA prior to COVID-19 restrictions, at the final sampling point, and the difference between these time points were entered into the model separately given that Spearman’s correlations indicated a relationship between these variables (R = −0.294 to 0.581; *p* < 0.005). For each ordinal regression performed, Nagelkerek pseudo-R-squared was reported and the test of parallel lines was used to confirm the assumption that the effect of the independents was the same for each level of the dependent variables.

Chi-squared analysis was performed to determine statistical differences in the frequency of response at each level of the COM-B model. This was then repeated to determine if frequencies differed between groups. When Chi-squared analysis indicated a significant effect of group, adjusted standardised residuals (ASR) were calculated to ascertain the nature of the between-group difference [34]. ASR of >1.96 or <−1.96 was used as a threshold to determine specific between-group differences [34]. When conducting Chi-squared analysis at a group level, on a small number of occasions at the lowest level of the model, the proportion of responses was too low to meet the sample size assumptions of the Chi-Squared test and in these instances, maximum likelihood Chi-square was determined [35]. Cramer’s V was calculated to determine the effect size.

## 3. Results

### 3.1. Physical Activity

**Figure 2:**
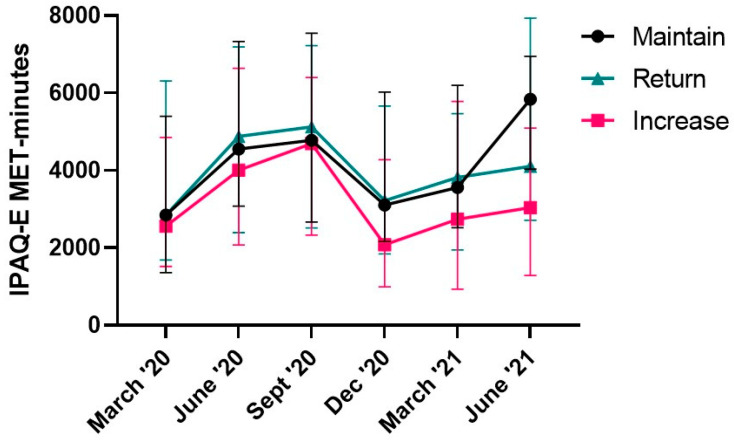
The influence of COVID-19 measures in the United Kingdom on the physical activity of individuals grouped by intended physical activity behaviour. (March ’20 represents Pre COVID-19 restrictions; Data presented as Median and interquartile range; Maintain *n* = 29; Return *n* = 29; Increase *n* = 38).

Self-reported PA did not differ between groups (Figure 2. *p* = 0.105; *η*^2^ = 0.047) and there was no group*time interaction (Figure 2. *p* = 0.471; *η*^2^ = 0.019). Self-reported PA was affected by time Figure 2. *p* < 0.001; *η*^2^ = 0.087). PA at March ’20 (pre-COVID-19) was lower than that measured in June ’20, September ’20 and June ’21 (Figure 2. *p* < 0.024; *g* > 0.30). PA measured in September ’20 was also greater than that measured in March ’21 and June ’21 (Figure 2. *p* < 0.007; *g* > 0.36). PA measured at March ’21 was lower than that at June ’20 (Figure 2. *p* = 0.046; *g* = 0.30) and June ’21 (Figure 2. *p* = 0.058; *g* = 0.29). Kruskal–Wallis tests confirm that there were no differences between groups at any time point (Figure 2. *p* > 0.238), other than in June ’21 (Figure 2. *p* = 0.024), where PA was greater in the Maintain compared to the Increase group (Figure 2. *p* = 0.02; *g* = 0.69).

Pre-COVID PA (R^2^ = 0.25; β = 8.271; SE(β) = 5.676; Wald χ^2^(1) = 2.123; *p* = 0.145), the PA measured at the final sample point (R^2^ = 0.001; β = −1.185; SE(β) = 5.234; Wald χ^2^(1) = 0.51; *p* = 0.821), and the differences between these measures (R^2^ = 0.30; β = −8.600; SE(β) = 5.304; Wald χ^2^(1) = 2.629; *p* = 0.105) was not associated with intended PA behaviour.

### 3.2. Perceived Barriers to Intended PA Target Behaviour

**Figure 3:**
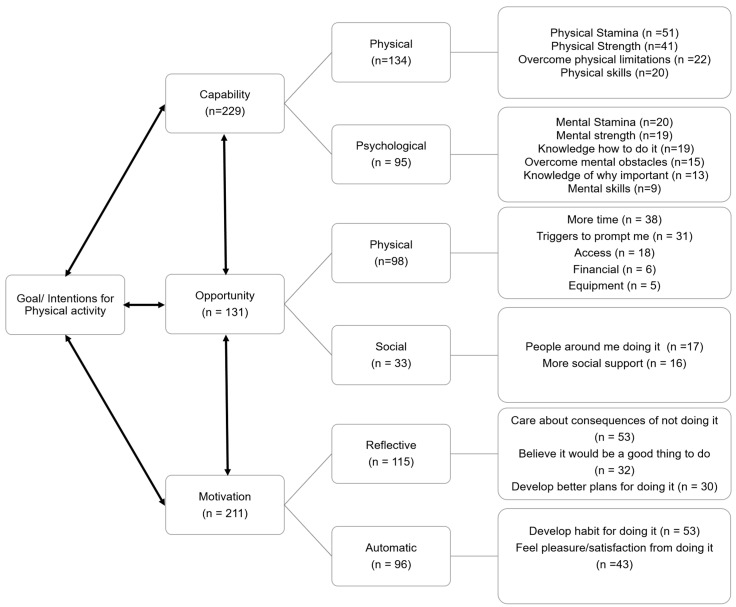
Perceived Capability, Opportunity and Motivation barriers to intended post COVID-19 restrictions PA behaviour.

The frequency of reported opportunity barriers to the intended PA behaviour was lower than barriers associated with capability and motivation (Figure 3. *X*^2^ (2, *n* = 571) = 28.595; *p* < 0.001; V = 0.224). Physical capability barriers were more frequently reported (Figure 3. *X^2^* (1, *n* = 229) = 6.642; *p* = 0.010; V = 0.170), where ‘physical stamina’ and ‘physical strength’ were most common (Figure 3. *X*^2^ (3, *n* = 134) = 20.209; *p* < 0.001; V = 0.388). There were no differences in the frequency of specific psychological capability barriers (Figure 3. *X*^2^ (5, *n* = 95) = 5.863; *p* = 0.320; V = 0.248). 

Physical opportunity barriers were more frequently reported (Figure 3. *X*^2^ (1, n = 131) = 32.252; *p* < 0.001; V = 0.496), where ‘more time’ and ‘triggers to prompt me’ were most common (Figure 3. *X*^2^ (4, *n* = 98) = 44.347; *p* < 0.001; V = 0.673). There was no difference in the frequency of specific social opportunity barriers (Figure 3. *X*^2^ (1, *n* = 33) = 44.347; *p* = 0.862; V = 0.095).

There was no difference between the frequency of reported reflective and automatic motivation barriers (Figure 3. *X*^2^ (1, *n* = 211) = 1.711; *p* = 0.191; V = 0.090). ‘Care about consequences of not doing it’ was more frequently reported than the other reflective motivation barriers (Figure 3. *X*^2^ (2, *n* = 115) = 8.470, *p* = 0.014; V = 0.271) and there was no difference in the frequency of reported automatic motivation barriers (Figure 3. *X*^2^ (1, *n* = 96) = 1.042; *p* = 0.307; V = 0.104).

### 3.3. Influence of Intended PA Target Behaviour on Perceived Barriers to PA

**Figure 4:**
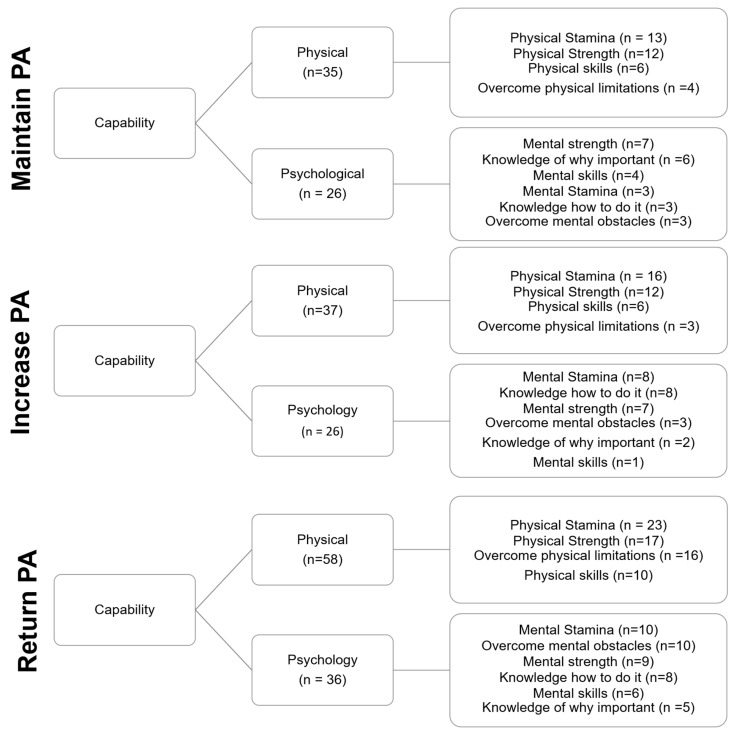
Perceived capability barriers grouped by intended PA target behaviour.

**Figure 5:**
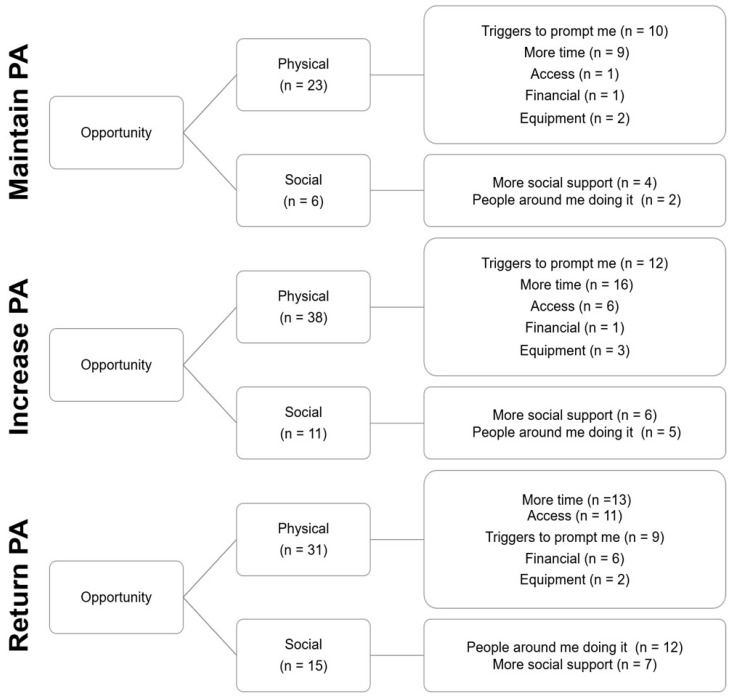
Perceived opportunity barriers grouped by intended PA target behaviour.

**Figure 6:**
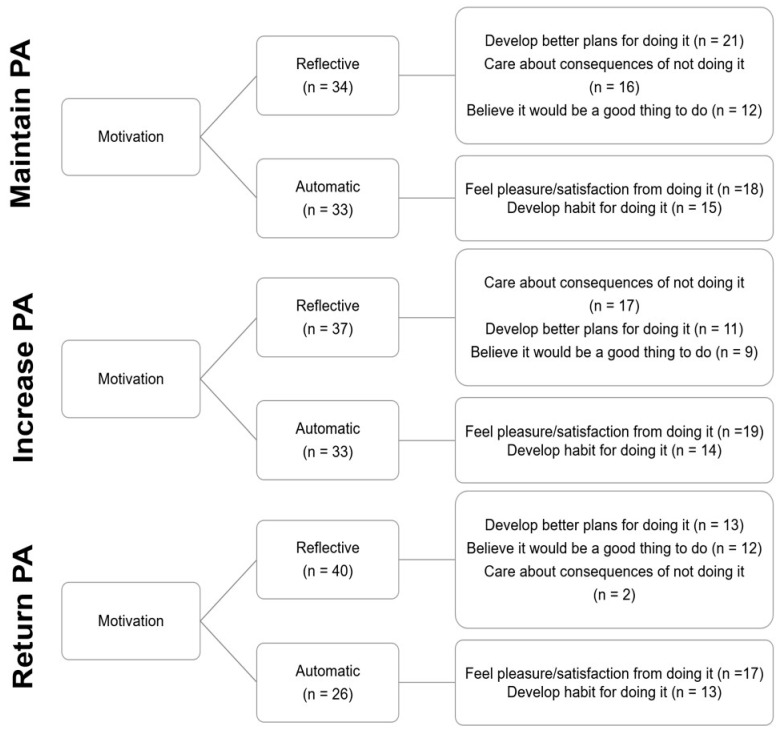
Perceived motivation barriers grouped by intended PA target behaviour.

There was a tendency for cited Capability, Opportunity and Motivation Barriers to differ between groups (Figure 4, Figure 5 and Figure 6. *X*^2^ (4, *n* = 545) = 8.490; *p* = 0.075; V = 0.88), with the return group citing a greater number of capability barriers (ASR = 2.1) but a lower number of motivation barriers (ASR = 2.0). 

The proportion of physical and psychological capability barriers did not differ between groups (Figure 4. *X*^2^ (2, *n* = 218) = 0.317; *p* = 0.853; V = 0.38). Physical stamina and physical strength were the most regularly cited physical barriers. The percentage of participants that cited each physical capability barrier did not differ between groups (Figure 4. *X*^2^ (6, *n* = 138) = 3.138; *p* = 0.791; V = 0.105). Specific psychological capability barriers were more varied between the groups, but were not statistically different (Figure 4. *X*^2^ (10, *n* = 103) = 4.156; *p* = 0.940; V = 0.137).

The proportion of cited Physical and Social opportunity barriers did not differ between groups (Figure 5. *X*^2^ (2, *n* = 124) = 1.797, *p* = 0.407, V = 0.120). The proportion of participants that cited each specific physical (Figure 5. *X*^2^ (8, *n* = 102) = 10.262, *p* = 0.247, V = 0.216) and social opportunity barrier did not differ between groups (Figure 5. *X*^2^ (2, *n* = 36) = 0.309, *p* = 0.857, V = 0.093).

The proportion of cited Reflective and Automatic motivation barriers did not differ between groups (Figure 6. *X*^2^ (2, *n* = 203) = 1.448; *p* = 0.485; V = 0.084). Furthermore, the proportion of participants that cited each specific Reflective (Figure 5. *X*^2^ (4, *n* = 113) = 4.734; *p* = 0.316; V = 0.135) or Automatic (Figure 6. *X*^2^ (2, *n* = 96) = 0.065; *p* = 0.968; V = 0.026) barrier did not differ between groups.

## 4. Discussion 

Given the importance of PA for healthy ageing [5], and in light of the well-reported effects of the COVID-19 pandemic on PA behaviour [8], during the initial period of COVID-19 recovery, the present study sought to provide important insight into the intended PA behavioural goals of older adults living in the UK, barriers to the intended PA behaviour and to understand to what extent these outcomes were influenced by self-reported PA across the time course of government-imposed restrictions. Our results indicate that although self-reported PA fluctuated across the time course of this study, there was no difference in the PA profiles between groups with different PA behavioural goals, other than at the final sampling point where the Maintain group completed more PA than the Increase group. Furthermore, self-reported PA was not associated with intended PA behaviour. The COM-B questionnaire indicated that capability and motivation factors were the most frequently cited barriers to the intended PA behaviour, which for the most part were equivalent across groups. However, individuals with the intention to return to pre-COVID PA behaviours demonstrated a tendency to cite a greater number of capability barriers, but a lower number of motivation barriers compared to other groups. These findings provide important information to stakeholders in devising the COVID-19 recovery public health policy for promoting healthy ageing and uses principles imbedded into the BCW [24], which should be considered in the design of future interventions.

As reported in our previous work [14], PA fluctuated over the time course of the study which was reflective of government-imposed restrictions in response to the COVID-19 pandemic and seasonal variation in PA, where adverse weather conditions have been shown to reduce PA in older adult populations [36]. On average, participants in the current study were more active than that reported generally for UK older adults [37], and may not have followed the general trends of a pandemic induced reduction in PA for older adults reported in other work (see review [8]). In support, Suzuki et al. [38] reported that whilst less active older adults saw a decline in PA during the early part of the pandemic, physically active older adults had a 47% increase in PA. Whilst the limited changes in PA over the course of the study likely represent adaptations to PA behaviours, these seemingly positive changes were not sufficient to offset a clinically meaningful reduction in perceived physical function [14]. This highlights the value of evaluating PA target behaviour and perceived barriers to achieving this outcome in the population studied, in order to develop targeted intervention strategies to mitigate negative effects on function. 

Seventy percent of the population sampled indicated the intention to maintain (30%) their current PA behaviour or return (40%) to their pre-pandemic PA behaviour. Based on the data in the present study, both intentions are unlikely to be sufficient to offset the clinically meaningful change in perceived physical function reported in this population, which has been further linked to fear of falling and reduced functional fitness and PA engagement [19,39,40,41]. Whilst a return to prior PA may intuitively be beneficial, there was limited difference in pre-pandemic PA and that of March ’21, where comparison between periods that negate seasonal variation is most appropriate for detecting change. As such, in the context of the current study, the return may refer to a return to PA behaviours rather than a change in frequency. However, a return to pre-COVID-19 PA behaviours may result in improved wellbeing and quality of life, particularly if the social benefits of PA are harnessed [42]. Similarly, despite the Maintain group completing more PA than the Increase group at the final sample point, the level of PA was similar to the season matched 12 month prior equivalent. Thus, maintaining PA behaviour is unlikely to be the most effective strategy to optimise the health benefits of PA for healthy ageing.

The transtheoretical model, which has been the basis of effective interventions to promote the adoption of healthy PA behaviours [22], suggests that health behaviour change involves cyclical progress through four stages until behaviour maintenance is achieved [43]. Identifying the stage of behaviour changes allows the targeted use of strategies and techniques to help promote transition to the next stage of the model [43]. With respect to the present study, the transition between pandemic induced changes in PA behaviour and the desired behaviour intention means that irrespective of PA ambition, many of the population sampled in this study fall between the pre-contemplation and the preparation stages (planning to make changes or making small changes in PA behaviour). Whilst the high level of PA in this population indicate that the value of PA is understood, understanding and overcoming the perceived barriers to the PA target behaviour and awareness-raising (consciousness-raising) of effective PA modalities are important steps in the transition to action (demonstrating new behaviour) and in maintaining (sustained change for at least six months) behaviour change. 

Data in the present study demonstrate a broad spread of perceived capability, opportunity and motivation barriers to the target PA behaviour, which are likely reflective of the individual needs of the older adult population. Whilst these results highlight a need to consider numerous and varied strategies to promote positive PA behaviour in the post-pandemic recovery, our findings indicate perceived capability and motivation barriers were cited more frequently than opportunity barriers. Having the ‘physical stamina’ and ‘physical strength’ to achieve the intended PA behaviour were the most frequently reported capability barriers. Such findings support previous literature where physical function has been shown to be a common barrier to PA in older adults [17,19]. The impact of COVID-19 on perceived physical function [14] is likely to compound these effects, where impaired perceived physical function has been linked to fear of falling and reduced functional fitness and PA engagement [19,39,40,41].

There was a much greater spread of perceived motivation barriers to the intended PA behaviour. With respect to reflective motivation ‘care about the consequences of not doing it’ was more frequently selected than other reflective motivation barriers and the need to feel pleasure and satisfaction related to engagement in the desired PA behaviour was a highly prevalent automatic motivation barrier. Both outcomes highlight a need to focus on the development of intrinsic motivation and self-determined extrinsic motivation for PA behaviour, which has previously been shown to distinguish older adults’ activity levels [44]. Furthermore, evidence supports the benefits of emphasising positive affect in the design of PA interventions for older adults [45,46].

With respect to automatic motivation, there was a high prevalence of barriers relating to habit formation. Evidence indicates that the relationship between habit and PA is bidirectional, which confounded by a dearth of evidence, has resulted in a lack of clarity with respect to whether habit predicts PA or vice versa [47]. Despite this, data suggest that PA is partially regulated by non-conscious processes such as habit formation [48], with several studies demonstrating a positive relationship between habit and PA [47]. As such, strategies to develop positive PA habits may be important for sustaining the intended PA behaviour and should be considered in the design of future interventions. However, despite studies that have targeted habit formation to improve PA, the habit development process is complex, timely, highly individual, with specific strategies not well reported the literature [47].

Although less frequently reported than capability and motivation barriers, there was a high prevalence of perceived opportunity barriers. Physical opportunity constraints focused on time and reminders that promote engagement in the target behaviour were most prevalent. Whilst providing prompting may be a useful tool in the PA habit development phase [49,50], time as a constraint to PA in older adults is prevalent [16,51] but not consistently reported in all older adult populations [18]. Whilst in previous literature, the constraint of time has been associated with care responsibilities to elderly parents and grandchildren [51], given the COVID-19 imposed restrictions, time may be further constrained by a desire to commit to hobbies and interests that could not be sustained over the initial period of the pandemic. Interestingly, unlike in previous work examining the barriers and facilitators to PA in older adult groups, barriers related to financial constraints and social opportunity were less frequently reported [18,52,53]. This outcome may be specific to the study population, who were deemed to be of high socio-economic status [54].

### 4.1. Application for Older Adult PA Interventions

Despite the population in the present study being highly active and the limited changes in quantity of PA over the course of the COVID-19 pandemic, the present work highlights a need for age-appropriate support and information from health care providers on how to safely and successfully maximise the benefits of PA. Obtaining information regarding effective PA strategy has been evidenced as a barrier to PA engagement for older adults [51]. Whilst this might seem intuitive for individuals that identified a desire to increase PA levels, awareness-raising (consciousness-raising) focusing on the most effective PA strategies to evoke health benefits would also be beneficial for the ‘maintain’ group given the decrease in perceived physical function over the study period that has previously been reported in this group [14].

Using the COM-B model to evaluate perceived barriers to the desired PA goal is advantageous given that strategies for intervention can be mapped directly to the Behaviour Change Technique Taxonomy (BCTT) [55]. As summarised in Table 1, the BCTT identifies that demonstration of effective PA behaviour, instruction on how to effectively perform the desired PA behaviour, and feedback and monitoring PA behaviour and its outcomes may be appropriate techniques to incorporate into future physical activity interventions in this population.

Whilst similar barriers were generally prevalent irrespective of the PA target behaviour, there was a tendency for those with the intention to return to their Pre-COVID-19 PA behaviours to cite a greater number of capability barriers, but a lower number of motivation barriers. Whilst this might underpin the focus on developing physical capability, it also more generally highlights a need to consider intervention design with respect to the different PA behaviour intentions of older adult groups.

Finally, these results highlight a need for researchers and health care providers to carefully consider how intervention success is monitored. Despite COVID-19 imposed government restrictions influencing PA behaviour, these findings demonstrate that PA is not a stable construct in older adults and long term assessment of healthy ageing should consider alternative healthy ageing assessments such as physical function, body composition, wellbeing and quality of life as a long term marker of success. 

### 4.2. Limitations and Future Direction

Although this study offers the first insight into the PA intentions of older adults and barriers to the target PA behaviour following a period of COVID-19 pandemic induced behaviour change, it is not without limitations. Despite an opportunistic approach to sampling and the desire to reach a diverse older adult population, individuals in the present study were highly active, shared similar ethnic backgrounds, and were of high socio-economic status [54]. As such, future work is needed to understand the intended PA behaviours and perceived barriers of further older adults groups to better reflect the UK older adult population.

Despite the benefits of the COM-B self-assessment questionnaire, future work is now needed to understand the perceived barriers of older adults with different PA intentions in further detail. Whilst the impact of COVID-19 on the PA levels of older adults has been well established [8], there is little information regarding the evolution of PA and the influence of the pandemic on the perception of PA in this age group. Such information would be particularly pertinent in the group sampled in this study given that PA was in the most part equivalent across groups and was reasonably well maintained. Furthermore, understanding more specifically PA evolution over the course of the pandemic, may prove important to facilitate intervention design in more vulnerable older adult groups.

## 5. Conclusions

Following the easing of government enforced COVID-19 restrictions, older adults sampled varied in their PA behaviour intentions. The desire to increase, return or maintain PA was not influenced by self-assessed PA measured longitudinally over the time course of the pandemic. Interestingly, in this highly active sample, PA did not differ between groups other than at the final sample point, where those with desire to maintain PA behaviour reported higher PA than those with the intention to increase PA. Irrespective of PA intention, the results of the present study in combination with our previous work demonstrating a reduction in perceived physical function during this time, highlights a need for awareness-raising (consciousness-raising) regarding effective PA strategies. Capability and motivation factors were the most frequently cited barriers to the intended PA behaviour, with our data more specifically highlighting a need to focus on physical strength and stamina, reflective and automatic motivation through training, persuasion, education, enablement and environmental restructuring. These findings provide important information to stakeholders in devising the COVID-19 recovery public health policy for promoting healthy ageing.

## Figures and Tables

**Table 1:** Outcomes of the COM-B Self Evaluation Questionnaire mapped to Behaviour Change Technique (BCT) Taxonomy and Key Behaviour Change Techniques.

Source of Behaviour	What Needs to Change	Proposed Intervention Functions	Proposed BCT Taxonomy	BCT
Physical Capability	Physical skills–strength and Stamina	Training (strength and stamina)	Comparison of behaviour Shaping knowledge Feedback and Monitoring	Demonstration of behaviour: Provide observable sample directly/indirectly Instruction on how to perform the behaviour: Advise on how to perform strength and stamina training Feedback on Behaviour: Monitor and provide informative or evaluative feedback on behaviour, e.g., form, F.I.T. (Frequency, Intesity and Time of PA) Feedback on outcomes of behaviour: Monitor and provide feedback on outcome of performance of behaviour, e.g., strength and stamina changes
Reflective Motivation	Beliefs about consequences–Believe it would be a good thing to do Goals–Develop better plans for doing it	Education Persuasion Enablement	Natural consequences Comparison of outcomes Goals and Planning Associations Feedback and Monitoring	Information about health consequences: Provide information about health consequences of performing behaviour Credible source: Present verbal or visual communication from a credible source Problem solving: Analyse factors influences on behaviour and generate and select strategies to overcome Action planning: Prompt detailed planning of performance Prompts and Ques: Introduce or define environmental or social stimulus with the purpose of prompting or cueing behaviour. Self-monitoring of behaviour: Establish a method for the person to monitor and record their behaviour(s)
Automatic Reflection	Reinforcement–Develop a habit for doing it Emotion–Feel pleasure and satisfaction	Training Environment Restructuring Persuasion	Repetition and substitution Natural consequences Shaping knowledge	Habit formation: Prompt rehearsal and repetition of the behaviour in the same context Habit reversal: Replace unwanted habit with alternative behaviour Monitoring of emotional consequences: Prompt assessment of feelings after attempts at performing the behaviour
